# Diversity and transmission of Aleutian mink disease virus in feral and farmed American mink and native mustelids

**DOI:** 10.1093/ve/veab075

**Published:** 2021-08-28

**Authors:** Jenni Virtanen, Andrzej Zalewski, Marta Kołodziej-Sobocińska, Marcin Brzeziński, Teemu Smura, Tarja Sironen

**Affiliations:** Department of Veterinary Biosciences, Faculty of Veterinary Medicine, University of Helsinki, Agnes Sjöbergin katu 2, Helsinki 00790, Finland; Department of Virology, Faculty of Medicine, University of Helsinki, Haartmaninkatu 3, Helsinki 00290, Finland; Polish Academy of Sciences, Mammal Research Institute, ul. Stoczek 1, Białowieża 17-230, Poland; Polish Academy of Sciences, Mammal Research Institute, ul. Stoczek 1, Białowieża 17-230, Poland; Faculty of Biology, University of Warsaw, ul. Miecznikowa 1, Warszawa 02-096, Poland; Department of Virology, Faculty of Medicine, University of Helsinki, Haartmaninkatu 3, Helsinki 00290, Finland; Department of Veterinary Biosciences, Faculty of Veterinary Medicine, University of Helsinki, Agnes Sjöbergin katu 2, Helsinki 00790, Finland; Department of Virology, Faculty of Medicine, University of Helsinki, Haartmaninkatu 3, Helsinki 00290, Finland

**Keywords:** AMDV, sequencing, evolution

## Abstract

Aleutian mink disease virus (AMDV), which causes Aleutian disease, is widely spread both in farmed mink and wild mustelids. However, only limited data are available on the role of wild animals in AMDV transmission and spread. Our aim was to shed light on AMDV transmission among wild mustelids and estimate the effect of intense farming practices on the virus circulation by studying AMDV prevalence and genetic diversity among wild mustelids in Poland. We compared AMDV seroprevalence and proportion of PCR-positive individuals in American mink, polecats, otters, stone martens, and pine martens and used the phylogenetic analysis of the NS1 region to study transmission. In addition, we used a metagenomic approach to sequence complete AMDV genomes from tissue samples. In eastern Poland, AMDV seroprevalence in wild mustelids varied from 22 per cent in otters to 62 per cent and 64 per cent in stone martens and feral mink, respectively. All studied antibody-positive mink were also PCR positive, whereas only 10, 15, and 18 per cent of antibody-positive polecats, pine martens, and stone martens, respectively, were PCR positive, suggesting lower virus persistence among these animal species as compared to feral mink. In phylogenetic analysis, most sequences from feral mink formed region-specific clusters that have most likely emerged through multiple introductions of AMDV to feral mink population over decades. However, virus spread between regions was also observed. Virus sequences derived from farmed and wild animals formed separate subclusters in the phylogenetic tree, and no signs of recent virus transmission between farmed and wild animals were observed despite the frequent inflow of farmed mink escapees to wild populations. These results provide new information about the role of different mustelid species in AMDV transmission and about virus circulation among the wild mustelids. In addition, we pinpoint gaps of knowledge, where more studies are needed to achieve a comprehensive picture of AMDV transmission.

## 1. Introduction

Aleutian mink disease virus (AMDV), species *Carnivore amdoparvovirus 1*, belongs to the genus *Amdoparvovirus* in family *Parvoviridae* (Shahrabadi, Cho, and Marusyk 1977; Bloom, Race, and Wolfinbarger 1980; Canuti, Whitney, and Lang 2015). AMDV causes high antibody titers, plasmacytosis, and immune complex disease (Aleutian disease, AD), with clinical signs ranging from subclinical to fatal. Signs include, for example, malaise, anorexia, neurological symptoms, renal failure, and reduced litter size in adults and pneumonia in mink kits. AD was first detected in American mink (*Neovison vison*) in 1956 but has since spread to all mink-producing countries and the wild (Aasted 1985; Bloom et al. 1994).

In addition to American mink, antibodies against AMDV or viral DNA have been found in several other carnivore species, including ferret (*Mustela putorius furo*), European mink (*Mustela lutreola*), pine marten (*Martes martes, Martes americana*), stone marten (*Martes foina*), polecat (*Mustela putorius*), stoat (*Mustela erminea*), skunk (*Mephitis mephitis*), otters (*Lutra lutra, Lontra canadensis*), raccoon (*Procyon lotor*), fox (*Vulpes vulpes*), bobcat (*Lynx rufu*s), lynx (*Lynx canadensis subsolanus*), and common genet (*Genetta genetta*) (Ingram and Cho 1974; Fournier-Chambrillon et al. 2004; Farid 2013; Knuuttila et al. 2015; Virtanen et al. 2020; Canuti et al. 2020a). In addition to AMDV, other amdoparvoviruses, such as skunk amdovirus (SKAV) (Canuti et al. 2017), red panda amdoparvovirus (RpAPV) (Alex et al. 2018), raccoon dog and fox amdoparvovirus (RFAV) (Shao et al. 2014), gray fox amdovirus (GFAV) (Li et al. 2011), Labrador amdoparvovirus 1 and 2 (LaAV-1 and -2) (Canuti et al. 2020), and red fox fecal amdovirus (RFFAV) (Bodewes et al. 2014), have also been found from carnivores. Many of these can also cause severe symptoms. Symptoms of AD have been described in American mink, ferret, and skunk (Henson et al. 1976; Porter, Porter, and Larsen 1982; LaDouceur et al. 2015), and it has been speculated that AMDV might be one of the reasons behind the decline of European mink populations (Fournier-Chambrillon et al. 2004). However, information about the influence of AMDV on native wild animal species and their health is limited. Even though reports of AMDV in humans are rare, its zoonotic potential has also been considered after studies reporting AMDV antibodies and DNA in exposed humans (Jepsen et al. 2009).

AMDV spread between and within countries and between farmed and wild animals has been studied with phylogenetic methods. These studies indicate that AMDV strains around the world are diverse and the virus transport occurs frequently between countries, even though country- and region-specific clusters are also detected (Ryt-Hansen et al. 2017; Virtanen et al. 2019; Prieto et al. 2020). AMDV has been introduced to mink farms in Poland from other countries in several different events, but the strains also show strong regional clustering due to spreading between farms and local outbreaks (Kowalczyk, Horecka, and Jakubczak 2019). Since AMDV can infect several animal species, wild animals are a possible vector transmitting the virus between different regions. However, comparisons between AMDV sequences from farmed and wild animals are limited and have given varying results. In Finland, AMDV sequences from feral mink were mixed with sequences from farmed mink in the phylogenetic tree, indicating at least some virus transmission between farmed and wild animals during the last couple of decades (Virtanen et al. 2019). On the other hand, AMDV sequences from wild and farmed animals in Poland have been in completely separate branches (Jakubczak et al. 2017). However, earlier studies have either been focusing on AMDV in farms or included a very limited number of sequences, and within-country variation has not been considered.

The aim of this study was to gain information about AMDV transmission between American mink and native mustelids in order to estimate the role of wild mustelids in virus transmission between geographical regions. We studied the prevalence of antibodies against AMDV and viral DNA in wild mustelids in Poland and used phylogenetic analysis to compare the virus strains found from wild mustelids and farmed mink, as well as between different geographical regions of Poland.

## 2. Materials and methods

### 2.1 Samples

**Figure 1.**
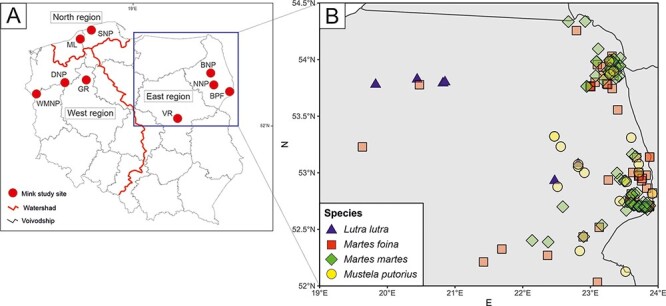
Sampling sites of feral mink (A) and other mustelids (B).

Samples from feral mink, farmed mink, otters, pine martens, stone martens, and polecats were collected between 2007 and 2018. Farmed mink were sampled from three farms in northwestern Poland. Martens, otters, and polecats were all collected in the eastern part of the country, and feral mink were collected at nine sites: Białowieża Forest (BPF), Biebrza National Park (BNP), Narew National Park (NNP), Vistula River (VR), Gwda River (GR), Drawa National Park (DNP), Warta Mouth National Park (WMNP), Słowiński National Park (SNP), and Modła Lake and surrounding area (ML; Fig. 1). The sites were grouped into three regions, which cover the areas of the main river basins: west—Oder River (WMNP, DNP, and GR); east—Vistula River (VR, NNP, DNP, and BF); and north—Baltic Sea tributaries (SNP and ML). The intensity of mink farming is highest in the western region, moderate in the northern region, and lowest in the eastern region (Zalewski et al. 2020). Wild mustelids were collected as roadkills, delivered by hunters, or acquired from eradication programs for nature protection plans. Carcasses were frozen and stored at −20°C before dissection, in which their sex was determined, and their hearts and spleen were collected.

**Table 1.** Prevalence of ELISA-positive and PCR-positive individuals of wild mustelid species in eastern Poland. Results are reported as percentages; 95 per cent confidence intervals are shown in brackets and absolute numbers (positive samples/all samples) in parenthesis. ELISA percentages are reported based on generalized linear model taking sex and season effect into account. PCR results are reported as percentages of positive individuals both from all samples (PCR 1) and ELISA-positive samples (PCR 2). PCR results were considered positive if at least one of the two PCRs that were used was positive.

Species	ELISA	PCR 1	PCR 2
*N. vison*	64 [60–68] (396/637^a^)	NA	96 [87–100] (52/54^b^)
*M. putorius*	48 [26–69] (9/19)	5 [0–14] (1/19)	11 [0–29] (1/9)
*M. martes*	35 [24–48] (27/63)	6 [0–12] (4/63)	15 [1–28] (4/27)
*M. foina*	62 [49–74] (40/61)	11 [4–21] (7/61)	18 [6–30] (7/40)
*L. lutra*	22 [5–58] (2/9)	0 (0/9)	0 (0/2)

a ELISA results from feral mink are from a previous publication [26].

b Only a subset of ELISA-positive feral mink was included in PCR analysis.

**Figure 2.**
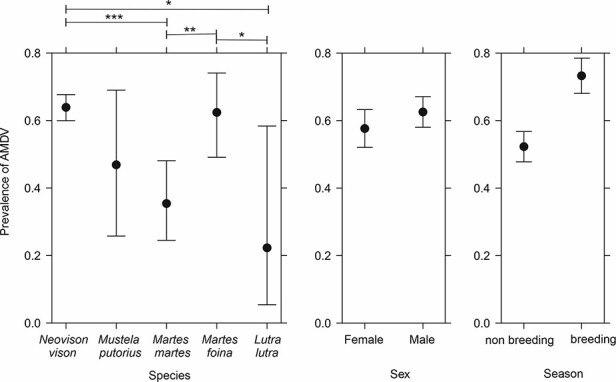
AMDV antibody prevalence in feral American mink and wild native mustelids in eastern Poland in different species, sex, and season. The confidence intervals and between-group differences were assessed using generalized linear model. Statistically significant differences are marked with *(*P* < 0.05), **(*P* < 0.005), and ***(*P* < 0.001) and 95 per cent confidence intervals are included in the pictures.

### 2.2 Serological studies

Mink samples (originally consisting of 1153 farmed and feral mink) had already been tested for AMDV antibodies with enzyme-linked immunosorbent assay (AMDV-VP2-ELISA) in our previous study (Zalewski et al. 2020). Due to the large number of samples, a subset was selected for further analysis by PCR. This includes all ELISA-positive farmed mink and 20 ELISA-positive feral mink from each site picked with simple random sampling without replacement. If there were less than 20 positive samples per region, all of them were included in the study. Samples from other mustelids were first studied for AMDV antibodies as described earlier (Knuuttila et al. 2009; Zalewski et al. 2020), and all ELISA-positive animals were subjected to PCR and sequencing. Detailed data about the animals are shown in the supplementary material (Supplementary Table S1).

### 2.3 Sanger sequencing

DNA was extracted from the heart or spleen of the selected farmed and feral mink and all ELISA-positive martens, polecats, and otters with NucleoSpin Tissue kit (Macherey-Nagel) using standard protocol for tissue samples. Partial AMDV sequences covering the nt 578–951 (partial nonstructural protein 1, NS1) and nt 1662–2302 (partial nonstructural proteins 1 and 2) (sites are according to AMDV-G (M20036.1) throughout the manuscript) were amplified with pan-AMDV- and pan-AMDO-PCRs as described earlier (Knuuttila et al. 2015; Virtanen et al. 2019) and positive results were confirmed with Sanger sequencing. PCR amplicons were purified for sequencing by adding 0.5 μl of Exonuclease I and 1 μl of FastAP Thermosensitive Alkaline Phosphatase (Thermo Scientific) to 5 µl of each PCR product and incubating them at 37°C for 45 min and 85°C for 15 min.

### 2.4 Whole-genome sequencing

A protocol set up for fecal samples by Conceição-Neto at al. (Conceicao-Neto et al. 2015) was modified to sequence AMDV from tissues. Approximately 50 mg of tissue was cut into small pieces with a scalpel, put in 500 µl of Dulbecco’s phosphate buffered saline + 0.2 per cent bovine serum albumin, and homogenized with MagnaLyzer without beads at 7000 rpm for 3 × 45 s. Samples were centrifuged at 17,000 *g* for 3 min and 350 µl of supernatant was filtered through a 0.8-μm filter with polyethersulfone membrane (Sartorius) at 17,000 *g* for 1 min.

Samples were then incubated at 37°C for 2 hours with a mixture containing 18.9 µl of 20× buffer (1 M Tris, 100 mM CaCl2, and 30 mM MgCl2, pH = 8), 5.4 μl of benzonase (Millipore), and 2.7 μl of micrococcal nuclease (New England Biolabs). Straight after nuclease treatment, DNA was isolated with a NucleoSpin Tissue kit (Macherey-Nagel) using support protocol for viral DNA from blood samples. Elution volume was decreased to 50 µl to increase DNA concentration.

As AMDV is a single-stranded DNA (ssDNA) virus, double-stranded DNA (dsDNA) was removed with a reaction that contained 16 µl of extracted DNA, 2 µl of dsDNase (Thermo Scientific), and 2 µl of 10× dsDNA buffer and was incubated at 37°C for 2 min. The reaction was purified with RNAClean XP beads (AGENCOURT) according to the manufacturer’s instructions.

DNA was amplified with the Complete Whole Transcriptome Amplification Kit (Sigma) according to the modified version of kit instructions described by Conceição-Neto at al. (Conceicao-Neto et al. 2015). Reactions were purified with PCR purification kit (GeneJet) or SPRIselect beads (Beckman Coulter), and DNA concentration was measured with Qubit using dsDNA HS Assay Kit (Thermo Scientific). Sequencing libraries were prepared using Nextera XT DNA Library Preparation kit or Nextera DNA Flex Library Prep kit (Illumina) according to the manufacturer’s instructions. The libraries were sequenced using v3 600 cycles sequencing kit and Illumina MiSeq.

### 2.5 Data analysis

Variation of AMDV antibody prevalence in eastern Poland was analyzed with the general linear model with a binomial family and three explanatory variables: species, sex, and season (breeding (February–August) and nonbreeding (September–January)). All pine and stone martens, otters, polecats, and feral mink from eastern Poland (Zalewski et al. 2020) were included in the analysis.

For the analysis of partial genomes (nt 578–951 and 1662–2302, as explained above), poor-quality sequences were first removed from the dataset. A collection of previously published AMDV sequences, including all published sequences from Poland and representative sequences from other countries, were retrieved from GenBank and included in the analysis. Due to the large amount of publicly available sequences, the global reference sequences for the nt 578–951 were selected based on a previously published phylogenetic tree containing all available sequences (Virtanen et al. 2019). For the nt 1662–2302, all AMDV sequences with at least 70 per cent query coverage published in GenBank by January 2021 were initially selected for a neighbor-joining tree that was used to select a set of sequences for the final phylogenetic analysis. The sequences were aligned using the ClustalW (Thompson, Higgins, and Gibson 1994) algorithm implemented in MEGA6 (Tamura et al. 2013). The best-fit evolutionary model was selected using the maximum likelihood method implemented in MEGA6. All the sequences from GenBank are listed in Supplementary Table S7. Correlations between genomic and geographical distances were estimated with Mantel test.

All alignments were analyzed for recombination with the programs RDP (Martin and Rybicki, 2000), GENECONV (Padidam, Sawyer, and Fauquet 1999), BootScan (Salminen et al. 1995), Max-Chi (Smith 1992), Chimaera (Posada and Crandall 2001), SiScan (Gibbs, Armstrong, and Gibbs 2000), and 3Seq (Boni, Posada, and Feldman 2007) implemented in the RDP4 or RDP 5 packages (Martin et al. 2015) using the highest acceptable *P*-value of 0.05. The recombinant sequences that were detected by at least four programs were excluded from phylogenetic analysis. Mean distances between and within groups were calculated with MEGA6 using *P*-values and pairwise removal of missing sites.

**Figure 3.**
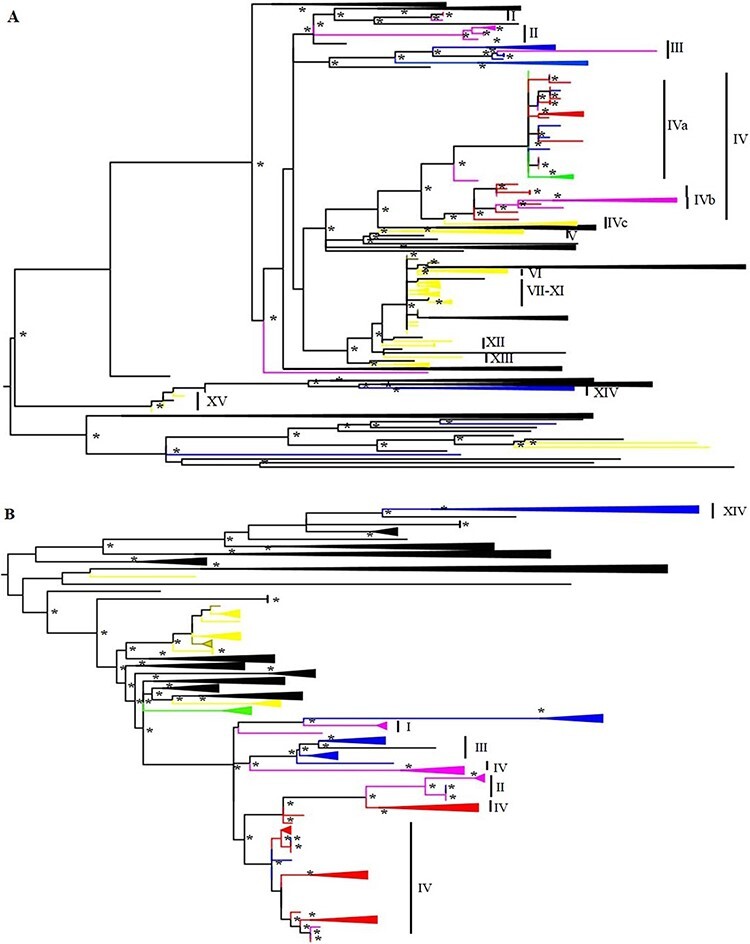
Phylogenetic trees based on nt 578–951 (A) and 1662–2302 (B). Boostrap values above 70 are indicated with * and substitution models are TVM + F + R4 (A) and TPM3 + F + R4 (B). Clades that include AMDV strains from a single geographical region have been collapsed and color coded with red (western Poland), pink (northern Poland), blue (eastern Poland), green (mustelids other than mink), yellow (strains from Polish farmed mink from previous studies), and brown (strains from Polish farmed mink sequenced in this study).

The phylogenetic trees were constructed with the maximum likelihood method implemented in IQ-TREE multicore version 2.1.2 (Nguyen et al. 2015) (Fig. 3 and Supplementary Fig. S1) using ModelFinder (Kalyaanamoorthy et al. 2017) and ultrafast bootstrapping (Hoang et al. 2018). In addition, a phylogenetic tree with molecular clock (Supplementary Fig. S2) was constructed from nt 578–951 with BEAST 1.8.2 (Drummond et al. 2012), Tracer v1.6 (Rambaut et al. 2014b), and FigTree v1.4.2 (Rambaut 2014a) using a 20,000,000 as chain length, Hasegawa-Kishino-Yano model (HKY + G) as an evolutionary model, lognormal relaxed clock as a clock model, and Bayesian skyline as tree prior. Effective sample size values were checked to be over 100.

For the construction of complete or nearly complete AMDV sequences, the raw next generation sequencing (NGS) reads were quality-filtered, de novo assembled, and annotated using Trimmomatic, Megahit, and SANSparallel programs, respectively, implemented in Lazypipe pipeline (Bolger, Lohse, and Usadel 2014; Li et al. 2015; Somervuo and Holm 2015; Plyusnin et al. 2020) using default parameters. In case more than one overlapping AMDV contig was found, these contigs were combined manually using AMDV-G (M20036.1) as reference. The raw sequence reads were then reassembled to the consensus sequence using the Bowtie2 (Langmead and Salzberg 2012) algorithm implemented in UGENE software (Okonechnikov, Golosova, and Fursov 2012). The sequences were analyzed together with all AMDV sequences with complete coding regions published in GenBank by 23 March 2021 and aligned in MEGA6 using Muscle (Edgar 2004). To analyze the potential recombination events, a nonredundant dataset of 70 complete genomes was constructed from the original dataset by removing sequences that had less than 1 per cent p-distance to any other sequence in the dataset. Recombination events detected by at least five programs implemented in the RDP5 were considered. A phylogenetic tree excluding the possible recombinants was built with IQ-TREE as described above.

Sites under positive or negative selection in the region amplified by pan-AMDV-PCR (aa 145–244 of NS1) were assessed with four methods available online (www.datamonkey.org, last accessed 27 August 2021) (Weaver et al. 2018): single-likelihood ancestor counting, fixed effect likelihood, mixed effects model of evolution, and fast, unconstrained Bayesian approximation for inferring selection (Kosakovsky Pond and Frost 2005; Murrell et al. 2012, 2013). Recombinant strains recognized by at least four programs of RDP were excluded as described above and only the sites that were recognized by at least two methods were accepted. The analysis was performed separately for all farmed strains from Poland (from this study and GenBank) and for strains of feral mink (from this study). It was also performed separately for feral strains from eastern and western Poland.

## 3. Results

### 3.1 AMDV seroprevalence in wild mustelids from eastern Poland

The prevalence of antibodies against AMDV among feral mustelids (637 feral mink (Zalewski et al. 2020), 63 pine martens, 61 stone martens, 19 polecats, and 9 otters) in eastern Poland are presented in Table 1 and Fig. 2. The prevalence was highest in feral mink (64 per cent, 396/637) and stone martens (62 per cent, 40/61) and smallest in otters (22 per cent, 2/9). Prevalence was 48 per cent (9/19) in polecats and 35 per cent (27/63) in pine martens. The difference was statistically significant between feral mink and otters (*P* = 0.026), feral mink and pine martens (*P* = 3.88e-05), pine martens and stone martens (*P* = 0.0038), and stone martens and otters (*P* = 0.040) (Supplementary Table S2). Prevalence was also significantly higher during breeding season compared to nonbreeding season (*P* = 2.90e-08). There was no significant difference between males and females (*P* = 0.18).

### 3.2 PCR results

In total, 98 per cent (112/114) of tested ELISA-positive feral mink samples and all 11 ELISA-positive farmed mink samples were positive in at least one PCR assay. Four of them were positive only with pan-AMDV-PCR and 12 only with pan-AMDO-PCR. When only feral mink from eastern Poland, where other species are collected from, are considered, 96 per cent (52/54) were positive in PCR. However, only up to 18 per cent of ELISA-positive martens, polecats, and otters were also PCR positive (Table 1). Sequencing confirmed 12 AMDV-positive samples from mustelids other than mink: 2 of those with both PCRs, 5 only with pan-AMDV-PCR and 5 only with pan-AMDO-PCR. One of the positive animals was a polecat, seven were stone martens, and four were pine martens.

### 3.3 Phylogenetic analysis of partial AMDV genomes

After excluding poor-quality sequences, phylogenetic analysis for the nt 578–951 was conducted with 87 sequences from this study (78 feral and 5 farmed mink sequences, 1 polecat sequence, and 3 stone marten sequences) and selected sequences from GenBank (Fig. 3A). Analysis for the nt 1662–2302 included 104 sequences, 102 of which were from mink (4 farmed mink and 98 wild mink), 1 from pine marten, and 1 from stone marten (Fig. 3B). No recombination was detected in either of the alignments. Trees have been simplified for the sake of clarity, and strains from different parts of Poland have been color coded. To ease the analysis and comparing of the trees, sequences from Poland from this study and GenBank were named as subclusters I–XV according to phylogenetic clustering based on the nt 578–951 so that each cluster only contains sequences from Poland (Fig. 3A and Supplementary Fig. S1A), excluding 151/NV/BNP/2010 and 1049/NV/NNP/2014 and several farm sequences that did not form specific clusters. Due to the large number of sequences and sequence diversity, cluster IV was further divided into subclusters a–c based on three major clades included in IV. Full phylogenetic trees with strain names and the tree including the molecular clock based on nt 578–951 are included in the supplementary material (Supplementary Figs S1 and S2).

Most AMDV strains from Polish farmed mink sequenced in this study grouped together with sequences derived from mink farms of Greater Poland Voivodeship and with other sequences from Polish farms that lack more specific location information. The strain 15/NV/Farm/2009 was the only exception as it clusters together with the sequences from wild mustelids from northwestern Poland (SNP and DPN). The time to the most recent common ancestor (tMRCA) of this clade was estimated to be 11 years (95 per cent highest posterior density (HDP) 8–15 years). Farm-derived sequences from eastern Poland (XVc) (Kowalczyk, Horecka, and Jakubczak 2019) are most closely related to a cluster of wild mustelid-derived strains from northwestern Poland (XVb), although with long branch length and estimated tMRCA of 58 years (95 per cent HDP 31–92 years). All the other strains from farmed animals formed clusters separate from wild mustelid-derived strains and were more closely related to AMDV strains derived from farmed mink in other countries.

Strains from feral mink form several separate clusters, most of which have a clearly identifiable main geographical region (Fig. 3A and Supplementary Fig. S1A). An exception to this is cluster XVa (tMRCA 29 years, 95 per cent HDP 16–47 years) that contains a mixture of sequences from northern, eastern, and western Poland, and no dominant region can be identified. Occasional mixing of individual sequences of feral mink from different geographical regions was noted in most other clusters as well, for example, 1370/NV/BPF/2016 from eastern Poland clusters with strains from northern Poland in both trees (II). There are also incongruencies between the tree topologies based on the two genomic regions, as several sequences are placed differently in the two trees, for example, the strain 215/NV/DPN/2011 clusters with other sequences from western Poland in the phylogenetic tree based on the nucleotides 1662–2302, while clustering with sequences from northern Poland in the tree based on the nt 578–951 (I).

The sequences 500/MF/2015 and 161/MF/2010 from stone martens grouped together with sequences from feral mink from WMNP, BNP, and SNP (XVa, identity 99.2 per cent, tMRCA 15.35 years, 95 per cent HDP 10–22 years), and the strain from stone marten 728/MF/2017 was most closely related to the sequence of feral mink from Finland (identity 95.1 per cent, tMRCA 34.51 years, 95 per cent HDP 11–63 years, nt 578–951) (Fig. 3A). Virus sequences from stone marten 191/MF/2012 and pine marten 506/MM/2015 were not closely related to other known Polish strains, and the closest resemblance was to strains from the Netherlands (similarity 98.0 per cent, 1662–2302 region) (Fig. 3B).

### 3.4 Genetic distance of AMDV within and between the study sites

Overall genetic mean distance was 7.5 per cent for the nt 578–951 region and 5.1 per cent for the nt 1662–2302 region when all sequences of this study were used, and 8.1 per cent and 5.2 per cent when other Polish sequences published in GenBank were also used. Within-group mean distances of clusters I–XV of nt 578–951 varied between 0.0 and 4.1 per cent (Table 2). Combined distance for clusters VI–IX was also calculated as they were members of the same tree branch that contained a lot of highly similar sequences from several countries and low boostrap values in branches separating them. When different geographical regions were compared, the genetic mean distance was highest in eastern Poland and lowest in western Poland. Genetic distances within study sites were highest in BNP and smallest in VR (Table 3 and Supplementary Fig. S3).

**Table 2.** Within-group mean distances of clusters I–XV of nt 578–951.

Cluster	Distance (%)	*N*	Group
I	0.44	3	Feral mink
II	0.74	5	Feral mink
III	4.11	13	Feral mink
IV	3.70	54	Farmed and feral mink
Iva	2.53	40	Feral mink
IVb	2.63	11	Feral mink
IVc	1.53	3	Farmed mink
V	1.60	6	Farmed mink
VI	1.83	3	Farmed mink
VII	0.47	8	Farmed mink
VIII	0.33	2	Farmed mink
IX	0.61	2	Farmed mink
VI-IX	1.01	15	Farmed mink
X	0.33	2	Farmed mink
XI	0.00	2	Farmed mink
XII	0.00	2	Farmed mink
XIII	0.87	3	Farmed mink
XIV	2.77	8	Feral mink
XV	0.00	13	Farmed mink

**Table 3.** Within-group mean distances of feral mink in Poland. Groups are based on geographical locations and have been sorted from largest to smallest based on nt 578–951.

	nt 578–951		nt 1662–2302	
Samples origin	Distance (%)	*N*	Distance (%)	*N*
Region				
East	9.77	31	5.48	44
North	5.73	19	4.34	18
West	3.39	26	2.25	35
Site				
BNP	11.17	12	4.60	16
NNP	8.58	12	5.48	19
BPF	7.36	2	3.44	4
SNP	5.23	13	4.51	12
ML	4.58	8	2.39	6
DNP	3.8	14	2.69	17
GR	2.12	3	2.87	3
WMNP	0.95	10	1.01	16
VR	0.27	5	0.18	7
Farm	6.82	67	2.25	18

**Figure 4.**
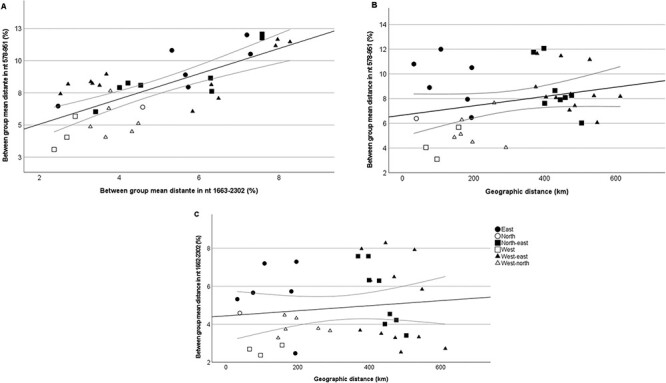
Comparison of genomic distances between nt 578–951 and nt 1662–2302 (A) and geographical and genomic distance between sites based on nt 578–951 (B) and nt 1662–2302 (C). Geographical distance is expressed as approximate distance between nine study sites and genetic distance as between group mean distance of nucleotide sequence. Ninety-five per cent confidence intervals of fit lines are included.

Genetic mean distances between the geographical regions varied between 5.15 and 9.47 per cent (nt 571–951) and 3.92 and 6.22 per cent (nt 1662–2302), being largest between east and north and smallest between north and west. Between the different study sites, the mean genetic distances ranged from 3.10 per cent to 12.0 per cent (nt 571–951) and 2.37 per cent to 8.28 per cent (nt 1662–2302) (Supplementary Table S3). Correlation between genetic and geographical distances between the study sites is visualized in Fig. 4. Observed correlations based on Mantel test were 0.104 (*P* = 0.042) in nt 578–951 and −0.026 (*P* = 0.68) in nt 1662–2038.

When farmed mink were compared to feral mink, mean genetic distance was highest in eastern region with low mink farming intensity (9.99 per cent in nt 571–951 and 7.08 per cent in nt 1662–2302) and smallest in western region with high farming intensity (8.10 per cent in nt 571–951 and 4.48 per cent in nt 1662–2302). Mean genetic distance within clusters of farm strains was less than 2 per cent in all cases, whereas in clusters of feral strains, it varied between 0 and 4.11 per cent (nt 571–951).

To study selection, we compared partial NS1 sequence codon-specific selection patterns between feral and farmed mink, as well as feral mink in eastern Poland (with small farming intensity) and feral mink in western Poland (with higher farming intensity). Altogether, 7 out of 100 codons were detected as positively selected in one or more study group and, respectively, 17 codons were detected as negatively selected (Supplementary Tables S4 and S5). Codons 159, 207, 209, and 214 of NS1 were positively selected and Codons 181, 211, and 213 were negatively selected in strains from both farmed and feral mink. Codon 210 was positively selected only in strains from farmed mink and Codon 234 only in strains from feral mink. Nine sites were negatively selected only in strains from feral mink and four sites only in strains from farmed mink.

### 3.5 Whole-genome sequencing

Complete or nearly complete AMDV genomes were sequenced with NGS from five samples that were selected based on their strong signals in pan-AMDV- and pan-AMDO-PCRs, clear Sanger sequence that did not indicate coinfection, and their different locations in phylogenetic trees from nt 578–951 and nt 1662–2302. No other ssDNA viruses were detected in the NGS data. The strains 158/NV/DPN/2010 and 869/NV/WMNP/2014 were sequenced from nt 3–4560 and 151–4560 with mean coverage values of 286 and 451 (Supplementary Fig. S4). In addition, nearly complete genomes of strains 11/NV/Farm/2009, 151/NV/BNP/2010, and 1049/NV/NNP/2014 were sequenced (excluding a few gaps ranging from a few to a couple of hundred nucleotides).

**Figure 5.**
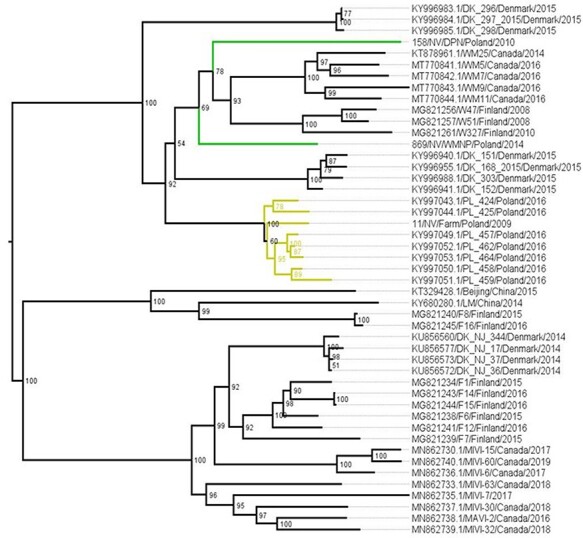
Phylogenetic tree on complete AMDV coding region sequence from this study and GenBank. The strains with recombination events suggested by at least four programs of RDP package were excluded from the dataset. Farm strains from Poland are marked with yellow and feral strains with green. Boostrap values are included next to the nodes, and the substitution model is GTR + I + G.

All 216 AMDV sequences with complete coding region from this study and GenBank were initially included for analysis. In order to construct a phylogenetic tree, the sequences showing evidence of recombination were excluded from the dataset. Consistently with the analyses based on partial genomes, the sequences from farmed and feral animals from Poland formed separate clusters (Fig. 5). Farm strains from Poland were most closely related to strains from Finland and Canada (between-group mean distance 5.9 per cent) and feral strains were most closely related to strains from Denmark (mean distance 4.7 per cent).

In total, recombination analysis suggested 19 recombination events (Supplementary Table S6). Out of the complete genomes sequenced in this study, 151/NV/BNP/2010 (breakpoint 1907) and 1049/NV/NNP/2014 (breakpoint 2988) showed potential recombination (Supplementary Figs S5 and S6). Based on RDP analysis, parental strains for 3ʹ end of the 151/NV/BNP/2010 were W181, W456, and W458 sequenced from feral mink from Finland, whereas parental strains for the 5ʹ end of the genome remained unknown. The similarity plot and bootscan analyses suggested the closest relatives to be 1049/NV/NNP/2014 (approx. first 500 nucleotides), KT329428/Beijing/China/2015 (nt 750–1250), and MG821246/F24/Finland/2017 (nt 1300–1800) (a representative of larger Finnish clade with less than 1 per cent sequence divergence). However, since several strains cluster together with 151/NV/BNP/2010 both before and after the suggested breakpoint and we have previously suggested that that the strains W181/W456/W458 may have recombinant origin (Virtanenet al. 2019), the recombination analysis of 151/NV/BNP/2010 remains uncertain. For 1049/NV/NNP/2014, no close relatives were found in the 5ʹ end, and this strain formed an outlier for a large group of sequences from Europe, China, and North America in the phylogenetic tree. After the suggested breakpoint, this strain formed an outgroup to a clade consisting of strains from China, Finland, and Poland. Given that the nonstructural protein-coding region of AMDV generally has more nucleotide diversity than the structural region (Nituch et al. 2012; Canuti et al. 2016), it is possible that the strain 1049/NV/NNP/2014 indeed is a highly divergent strain rather than a recombinant.

## 4. Discussion

Here, we report a comprehensive investigation of AMDV in wild mustelids in Poland providing information of virus circulation among wild animals, as well as between farmed and wild animals. Results of both antibody and PCR testing were combined to compare the virus persistence in different mustelid species and to shed light on their role in AMDV transmission.

### 4.1 Transmission of AMDV

Feral American mink has been introduced to Poland both through migration from eastern Europe in the 1980s (eastern Poland) and through farm escapees (western Poland). It has since formed several distinct populations that have later joined together as the populations have grown (Zalewski et al. 2010, 2011; Brzeziński et al. 2019). AMDV strains from feral mink mainly follow a similar pattern and form several geographical clusters, but there is also occasional mixing of viruses between different regions of the country (Fig. 3 and Supplementary Fig. S1). Virus strains from the northern, western, and eastern parts of Poland all form several clusters in the phylogenetic tree, indicating multiple introductions of AMDV into these regions possibly through farm escapees or past dispersal of wild animals.

Interestingly, the subcluster IVa contains a mixture of sequences from all the study regions instead of having one clearly dominant region like other clusters. The estimated tMRCA of branch IVa is smaller than in other clusters of feral strains (Supplementary Fig. S2), suggesting a rapid geographical spread of this virus lineage among feral mink. One explanation for the faster spread between all three regions that are located hundreds of kilometers apart and are separated by various habitat barriers could be virus transmission between farms, for example, through trade and then further transmission into the wild. There is only one farm strain in IVa, but details about how the farms are picked for previous studies are usually not explained in detail, and, therefore, sampling biases are possible. However, as mink farming in Poland is largely concentrated in the western and northern parts of the country, the hypothesis of spread through infected farms is less suitable when it comes to virus spread to eastern Poland where the farming intensity is significantly lower. Kowalczyk et al. also demonstrated that farm strains in eastern and western Poland were not similar around the same time frame these samples were collected (Kowalczyk, Horecka, and Jakubczak 2019). Notably, the tMRCA values based on short genomic fragment and potentially biased sampling should be considered as rough estimates that can be used to estimate whether the clusters have separated a few years or a few decades ago, and, therefore, the tMRCA of cluster IVa might actually be more than the estimated 29 years. Cluster IVa was not as clearly identifiable in nt 1662–2302, but even there, virus spread between all three regions was detected, even though the time frame for transmission could not be estimated. Different tree topologies can also be partly explained by the recombination that is common for AMDV or by different set of sequences in different trees. The genetic region of nt 1662–2302 is also more conserved than nt 578–951, which affects the phylogenetic analysis.

Virus strains from feral and farmed mink mostly form separate clusters. Most AMDV strains from farms are more closely related to strains from other European mink farms than strains from feral mink in Poland, and the tMRCAs span over several decades suggesting that (on the basis of the current data), it is unlikely that epidemics in farms originate from feral mink. Our results support the findings by Jakubczak et al. (2017) but are contradictory to the results from Newfoundland, Canada, where the AMDV strains from feral mink were found to be similar to the strains from local farms (Canuti et al. 2020b) and to the results of our previous study that indicated that AMDV antibody prevalence among feral mink was higher near the mink farms suggesting virus spread between farmed and feral mink populations in Poland (Zalewski et al. 2020). While the lack of genetic evidence on the (recent) transmission of AMDV between farmed and wild mustelids in Poland may be due to small or biased sampling, there are also alternative explanations, such as a very different virus epidemiology in the farms compared to the wild. Host population is denser in farms than in the wild, leading to easier virus transmission. Most farmed mink are also culled annually, and the new host generation is infected via horizontal or vertical transmission from a small population of breeding animals or via contaminated environment, whereas feral mink can have persistent infection for a longer time (Virtanen et al. 2019). Virus strains circulating in farms may also have changed since the introduction of the observed AMDV strains into the wild due to the constant control measures to eradicate the virus from infected farms followed by new introductions of different AMDV lineages, for example, through trade. In addition, pathogens like AMDV might affect farm escapees’ chances of survival and, therefore, reduce the probability of the establishment of continuous circulation of farm animal-derived AMDV lineages in the wild. A limitation in our analysis is that all the AMDV strains sequenced in this study come from a single farm since the animals from the two other farms were negative in ELISA screening and, therefore, the comparison between farmed and wild animals mostly relies on a large amount of sequence data from other studies (Ryt-Hansen et al. 2017; Kowalczyk, Horecka, and Jakubczak 2019). However, as all the samples have been collected from the same geographical regions during the same period of time, differences in the study designs are unlikely to explain the difference between the AMDV strains from farmed and wild animals in Poland. In conclusion, our study suggests that there is frequent virus transmission of AMDV between the wild mustelids. However, whether the transmission occurs through farm escapees, through migrating feral mink, through some yet unknown transmission route, or through all of these combined remains an open question.

When genetic distance and geographical distance between the study sites were compared, there was a positive correlation in nt 571–951 but not in nt 1662–2302. One possible reason for this may be the different substitution rate between the two genomic regions. While the analysis suggested general correlation between genetic distance in partial NS1 sequences and the geographical distance of sampling sites, there were also intriguing outliers from this trend, suggesting that, most likely, the geographical distance between the study sites is only one of many factors explaining the genetic diversity of AMDV. Genetic diversity was especially pronounced in eastern Poland, even when the study sites were close to each other. The difference between eastern as compared to northern and western Poland might be explained by geographical factors that were not taken into account in the analysis, different farming intensity, and different introduction routes of feral mink. As feral mink were most likely introduced to the eastern part of Poland through migration from other countries, the AMDV strains introduced first to this region may have already been more diverse than those introduced to the western and northern parts of the country, where many of the first feral mink originated from mink farms in the area. This, however, is difficult to prove as there is no sequence data available from mink farms around the time AMDV was introduced into the wild. Intense farming practices have been shown to speed AMDV evolution in the farms (Virtanen et al. 2019; Canuti et al. 2020a), but there is less data on how farming affects AMDV evolution in the wild. It could be that there is less virus spread between different sites because there are fewer farm escapees trying to find a habitat for themselves.

Since feral mink typically have a longer lifespan than farmed mink that are born late spring and culled during autumn, we hypothesized that infections in feral and farmed mink may pose different selection pressures to the virus. While there were differences between the groups (Supplementary Table S5), the direction (positive vs. negative) of selection was generally similar for each codon in all four study groups. Our results suggest that while the codon-specific selection pressures may be similar for both farmed and feral mink, the strength of the selection may differ between the groups. However, it should be noted that the datasets analyzed most likely do not represent the whole virus population harbored by a given study group due to the limited and potentially biased sampling, and the analysis was limited to a short region in NS1.

Recombination is frequent in AMDV and other parvoviruses (Shackelton et al. 2007; Ohshima and Mochizuki 2009; Wang et al. 2012; Canuti et al. 2016; Virtanen et al. 2019). Out of the five sequenced complete genomes, possible recombination was identified in the VP2 region of two strains. In addition, several sequences grouped differently in the phylogenetic trees based on the two different genomic regions, suggesting that there has most likely been recombination in other parts of the genome. This is supported by earlier studies that have identified a major breakpoint between these two regions (Canuti et al. 2016; Virtanen et al. 2019). Recombination complicates phylogenetic analysis and tracking of virus spread, as results depend on the region that has been used in the analysis. The optimal solution would be to analyze complete genomes; however, only few are available at the moment. In this study, 10–15 per cent of the already limited amount of complete coding regions published in GenBank were excluded from the phylogenetic tree due to recombination. This makes the dataset small and biased as it only contained sequences from a few studies (Fig. 4). Hence, we chose to use two short regions in the main phylogenetic analysis instead of just one, both supporting the same overall conclusions on the movement of AMDV across Poland.

### 4.2 AMDV prevalence in different mustelid species and their role in transmission

The percentage of antibody-positive individuals was high in stone martens and polecats, indicating that they are frequently infected by AMDV. The percentage was lower in otters, although the small sample size prevents any strong conclusions. The differences in the seroprevalence between the host species may be attributed to the different ecological niches of these animals. Contact between feral mink and otters may be limited since, most likely, mink avoid contact with bigger otters. However, the habitats of these two species are similar. Therefore, the AMDV seroprevalence among otters may be low because they are less easily infected, less in contact with mink, or simply because the sample size was small. The equal antibody prevalence of feral mink and stone martens may be related to the fact that stone martens inhabit rural areas, disperse far (up to 25 km) (Wereszczuk and Zalewski 2015), and might also easily get into the farms and be in contact with mink. The prevalence was smaller in pine martens, which prefer forests and avoid rural areas and possibly have less contact with farmed mink than stone martens (Wereszczuk and Zalewski 2015; Wereszczuk, Leblois, and Zalewski 2017). Polecats inhabit both rural areas and river and wetland habitats, and the amount of AMDV antibody-positive polecats was in between stone martens and pine martens. The higher prevalence during breeding season is most likely explained by the higher rate of contact between individuals and the possible effect of pregnancy on susceptibility to pathogens.

Regarding the PCR results, all sequences represented AMDV and not some other closely related amdoparvovirus even though pan-AMDO-PCR has been designed to also amplify other amdoparvoviruses. Even the sequences from samples that were positive with pan-AMDO-PCR and negative with AMDV-specific PCR represented AMDV. Ninety-six per cent of ELISA-positive feral mink from eastern Poland were positive in PCR, but in martens and polecats, only 10–20 per cent of ELISA-positive individuals were also positive in PCR. One possible explanation for this is that the viral loads in martens and polecats are too small for PCR to detect. Another explanation is that martens, polecats, and otters, unlike mink, have cleared the virus. Percentages of PCR-positive martens were similar to those reported in Canada by (Canuti et al. 2020b), who suggested that mink is a maintenance host to AMDV required for the persistence of the virus in the population even though spillover to other species is common. The high prevalence of antibody-positive individuals lacking persistent infection supports this hypothesis. This would also make it much less likely for martens, polecats, and otters to spread AMDV from one farm to another than mink as a lot smaller proportion of individuals carry the virus.

In conclusion, AMDV transmission is complex and is affected by several different factors, many of which are probably still unknown. In Poland, AMDV has been introduced to the wild mustelids in several separate events, and the virus forms region-specific clusters even though transmission between regions was also observed. Transmission between regions that are located hundreds of kilometers apart suggests that dispersion of wild mustelids has not been the only transmission route of AMDV in the wild in Poland. Compared to feral mink, a significantly lower proportion of antibody-positive native mustelids were also PCR positive, indicating that the virus is unable to replicate in them as well as it does in American mink. More studies are needed about the transmission of not only AMDV but also other viruses carried by wild animals, as some of these viruses may pose a threat to either endangered species, companion animals, or humans in contact with these animals.

## Supplementary Material

veab075_Supp

## Data Availability

All the sequences of this study have been deposited in GenBank under accession numbers MZ126964–MZ127162. All the other data are included in the manuscript or its supplementary material.
