# Supplementary material for: Diversity and transmission of Aleutian mink disease virus in feral and farmed American mink and native mustelids
Source: Virus Evol. 2021 Aug 28;7(2):veab075. doi: 10.1093/ve/veab075 (PMC8449508; doi:10.1093/ve/veab075)
Supplement: veab075_Supp [file veab075_supp.zip › Fig. S3.pptx]

## Slide 1
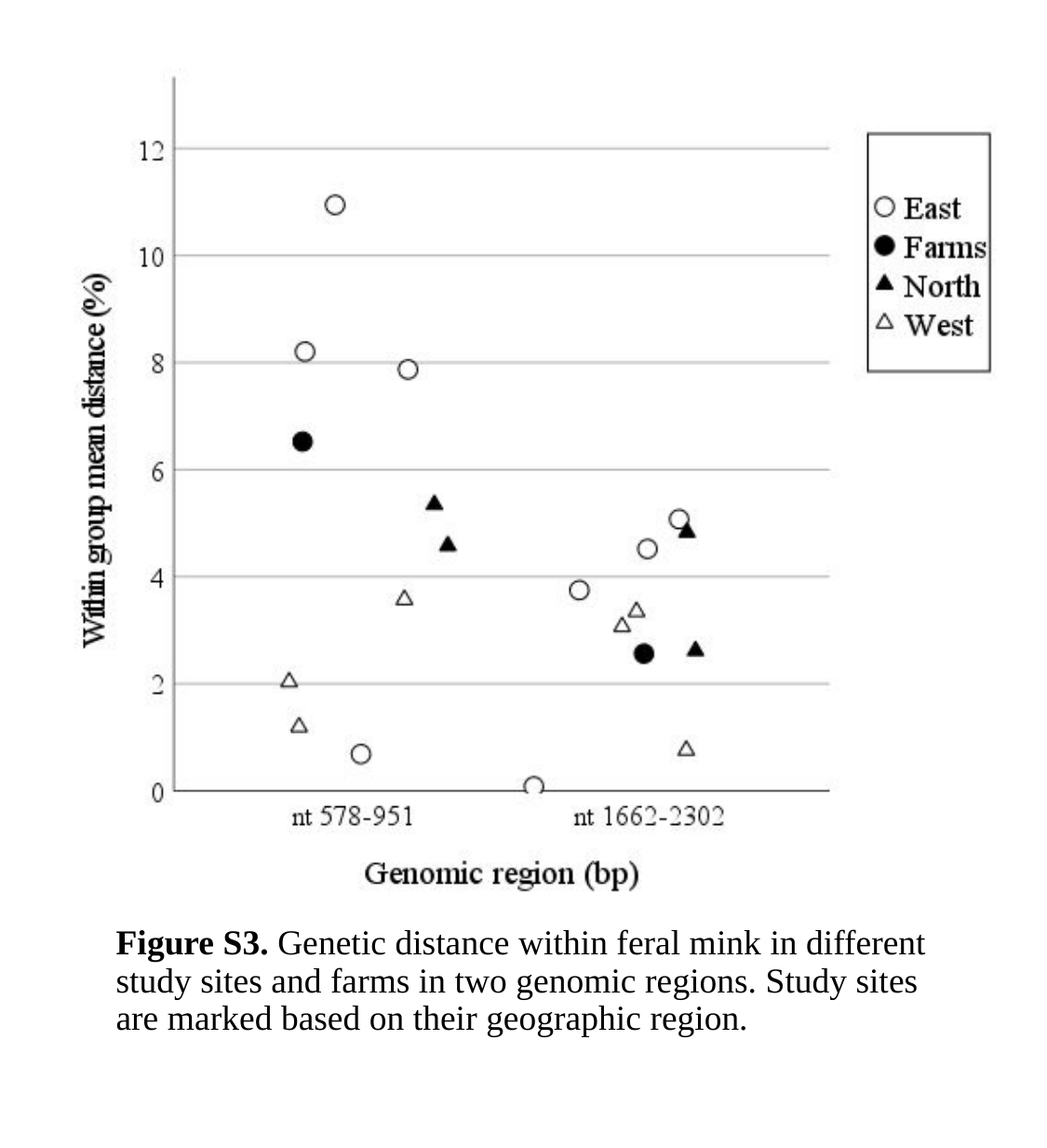

Figure S3. Genetic distance within feral mink in different study sites and farms in two genomic regions. Study sites are marked based on their geographic region.
