# Supplementary material for: Diversity and transmission of Aleutian mink disease virus in feral and farmed American mink and native mustelids
Source: Virus Evol. 2021 Aug 28;7(2):veab075. doi: 10.1093/ve/veab075 (PMC8449508; doi:10.1093/ve/veab075)
Supplement: veab075_Supp [file veab075_supp.zip › Fig. S6.pdf]

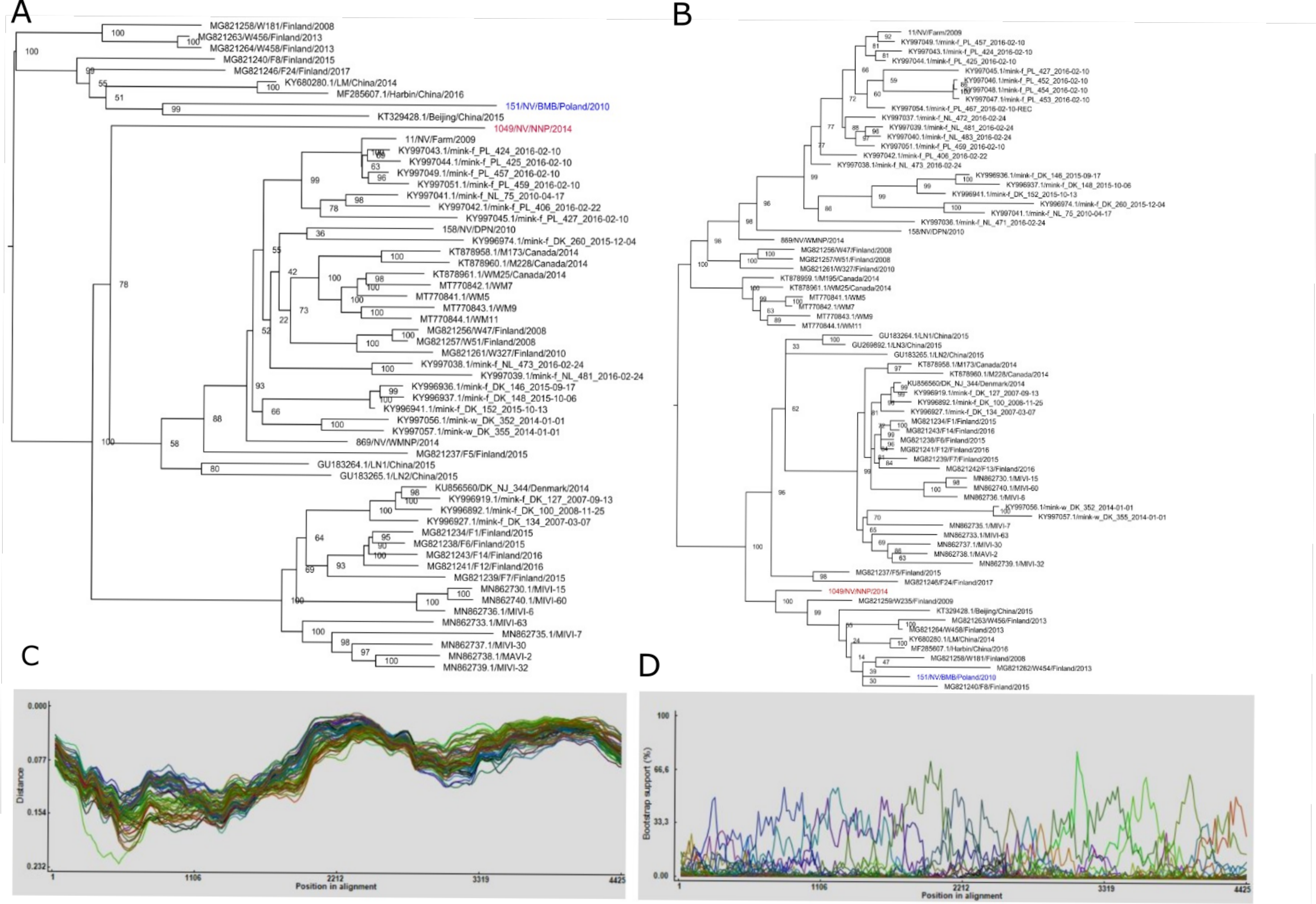

Fig S6. Recombination analysis of 1049/NV/NNP/2014 including IQ-trees from both sides of the recombination breakpoint 2988 (according to AMDV-G) (A and B), as well as simplot (C) and BootScan (D) analysis made by RDP5.
