# Supplementary figures and images for: Diversity and transmission of Aleutian mink disease virus in feral and farmed American mink and native mustelids
Source: Virus Evol. 2021 Aug 28;7(2):veab075. doi: 10.1093/ve/veab075 (PMC8449508; doi:10.1093/ve/veab075)

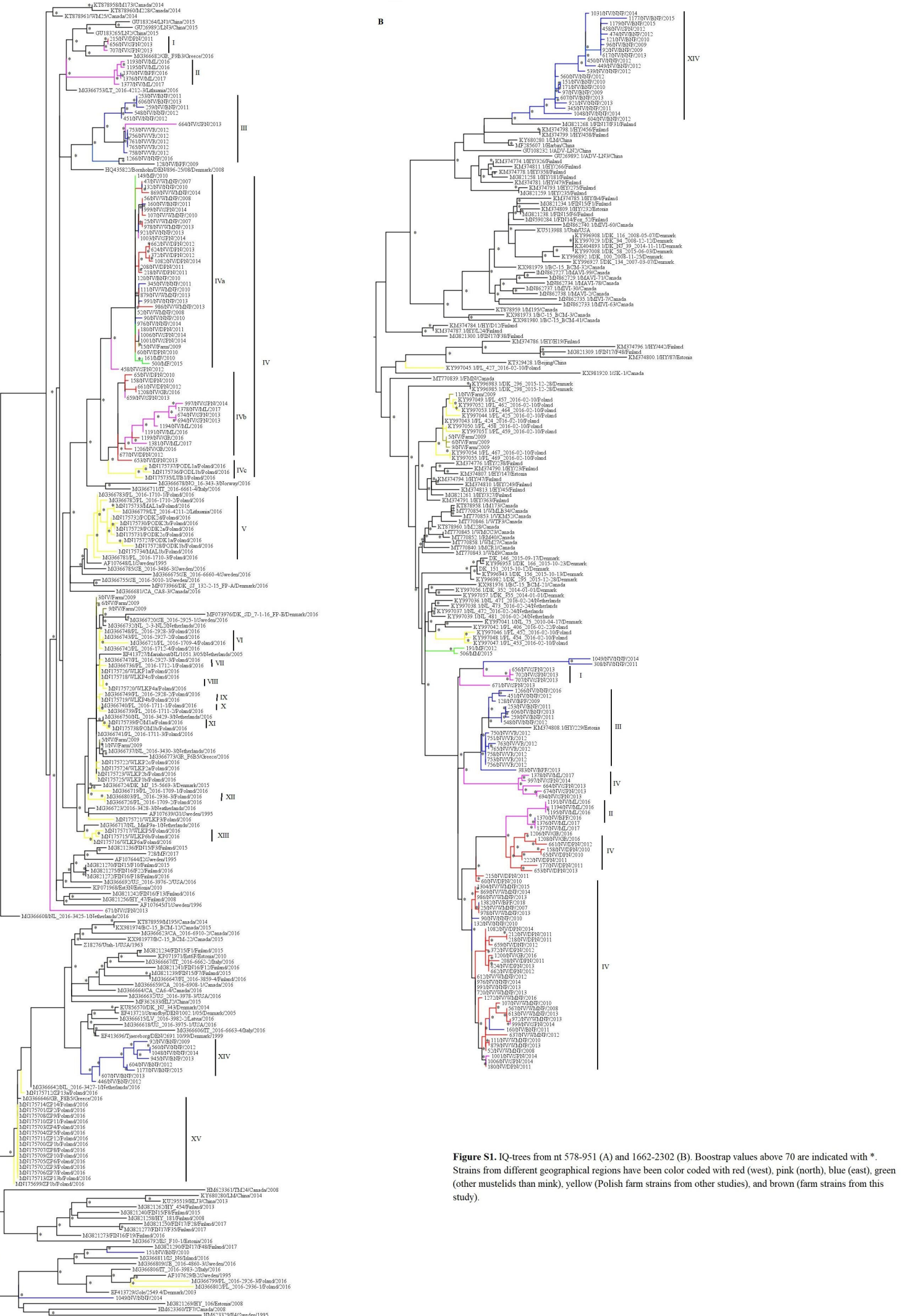

Supplement: veab075_Supp [file veab075_supp.zip › Fig. S1.pdf]

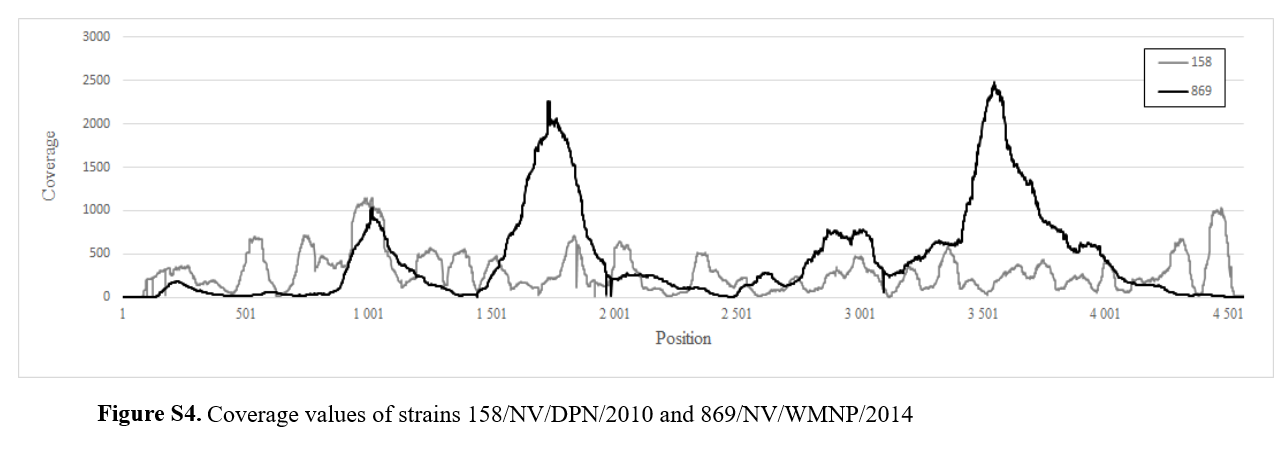

Supplement: veab075_Supp [file veab075_supp.zip › Fig. S4.png]
