# Supplementary material for: Diversity and transmission of Aleutian mink disease virus in feral and farmed American mink and native mustelids
Source: Virus Evol. 2021 Aug 28;7(2):veab075. doi: 10.1093/ve/veab075 (PMC8449508; doi:10.1093/ve/veab075)
Supplement: veab075_Supp [file Fig. S2.pdf › Fig. S2.pdf]

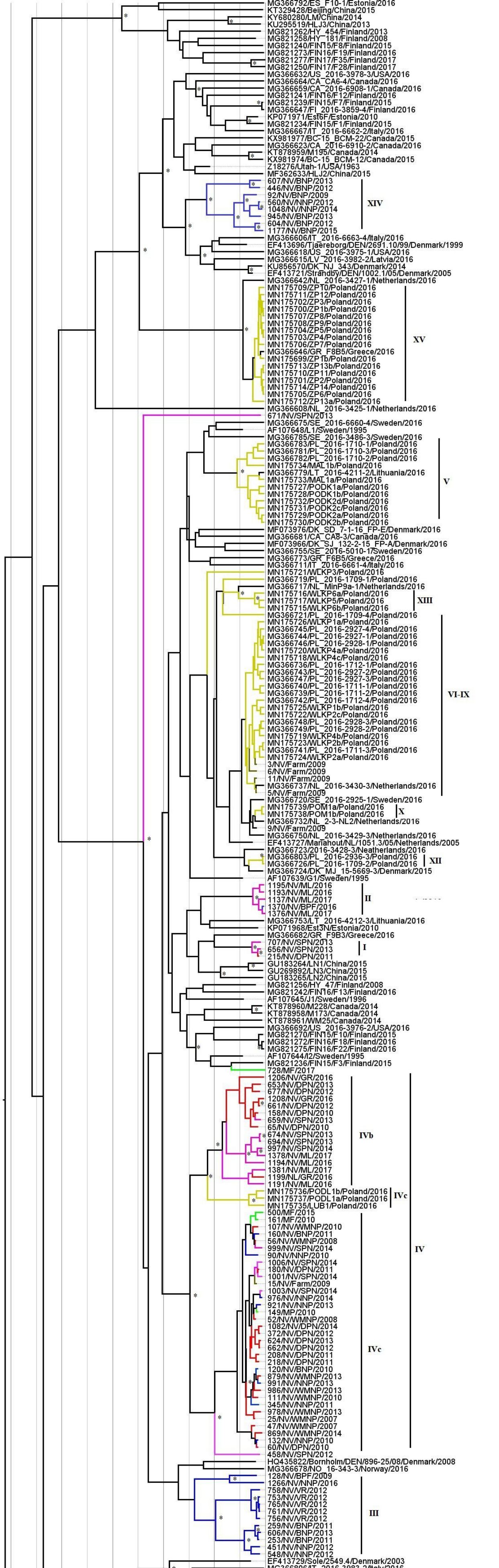

Figure S2. Bayesian phylogeny from nt 578-951 with molecular clock analysis. Posterior probabilities above 0.9 are indicated with * and timescale (one tick indicating 25 years) is included. Strains from different geographical regions have been color coded with red (west), pink (north), blue (east), green (other mustelids than mink), yellow (Polish farm strains from other studies), and brown (farm strains from this study).
